# Expression and Function of Serotonin 2A and 2B Receptors in the Mammalian Respiratory Network

**DOI:** 10.1371/journal.pone.0021395

**Published:** 2011-07-18

**Authors:** Marcus Niebert, Steffen Vogelgesang, Uwe R. Koch, Anna-Maria Bischoff, Miriam Kron, Nathalie Bock, Till Manzke

**Affiliations:** 1 Department of Neuro- and Sensory Physiology, Georg-August-University of Göttingen, Göttingen, Germany; 2 German Research Council Research Center for Molecular Physiology of the Brain, Göttingen, Germany; 3 Department of Child and Adolescent Psychiatry, Georg-August-University of Göttingen, Göttingen, Germany; University of Houston, United States of America

## Abstract

Neurons of the respiratory network in the lower brainstem express a variety of serotonin receptors (5-HTRs) that act primarily through adenylyl cyclase. However, there is one receptor family including 5-HT_2A_, 5-HT_2B_, and 5-HT_2C_ receptors that are directed towards protein kinase C (PKC). In contrast to 5-HT_2A_Rs, expression and function of 5-HT_2B_Rs within the respiratory network are still unclear. 5-HT_2B_R utilizes a Gq-mediated signaling cascade involving calcium and leading to activation of phospholipase C and IP3/DAG pathways. Based on previous studies, this signal pathway appears to mediate excitatory actions on respiration. In the present study, we analyzed receptor expression in pontine and medullary regions of the respiratory network both at the transcriptional and translational level using quantitative RT-PCR and self-made as well as commercially available antibodies, respectively. In addition we measured effects of selective agonists and antagonists for 5-HT_2A_Rs and 5-HT_2B_Rs given intra-arterially on phrenic nerve discharges in juvenile rats using the perfused brainstem preparation. The drugs caused significant changes in discharge activity. Co-administration of both agonists revealed a dominance of the 5-HT_2B_R. Given the nature of the signaling pathways, we investigated whether intracellular calcium may explain effects observed in the respiratory network. Taken together, the results of this study suggest a significant role of both receptors in respiratory network modulation.

## Introduction

Immunohistochemical and electrophysiological studies carried out over the previous twenty years have provided considerable evidence that serotonin (5-HT) released from caudal medullary raphé nuclei modulates respiratory network discharges in bulbar and spinal regions [1]–[8]. Subsequent research set out to determine which subtypes of 5-HT receptors (5-HTRs) are operative as pharmacological targets for a potential therapy to treat centrally caused breathing disturbances [9]–[17]. Those studies revealed that 5-HT_1A_, 5-HT_2A/C_, and 5-HT_4(a)_ receptors modulate respiratory network discharge properties. These receptors represent only a fraction of the 5-HTR subtypes that modulate excitability of CNS neurons through various signaling pathways.

Amongst the 5-HTR family 5-HT_2_Rs include 5-HT_2A_, 5-HT_2B_, and 5-HT_2C_R isoforms that couple preferentially to G_q/11_-proteins. The resulting activation of phospholipase C (PLC) increases hydrolysis of inositol phosphates and elevates cytosolic Ca^2+^ [18], [19]. 5-HT_2_Rs are located post-synaptically [20]–[22], and there is evidence that they modulate neurotransmission at various central and peripheral synaptic sites [23], [24].

5-HT_2A_Rs stimulate PLC, leading to activation of protein kinase C (PKC), and increased excitability in bulbar respiratory neurons [25]–[27]. Earlier studies demonstrated PKC pathway-mediated modulation of the respiratory pattern [26] and excitation of respiratory neurons by activation of 5-HT_2A_Rs [25], [27]. Beside direct modulation of the respiratory motor pattern, 5-HT_2A_Rs may have a key role in the induction of long-term facilitation of phrenic nerve activity in response to intermittent hypoxia [28]–[31].

5-HT_2B_Rs have been implicated in anxiety, schizophrenia, autism, migraine, and spreading depression [32]. In addition, 5-HT_2B_R-dependent serotonin uptake influences the plasma serotonin level [33]. 5-HT_2B_Rs are also important regulators of embryonic development; inactivation of the 5-HT_2B_R gene leads to partial embryonic and early neonatal death in mice [34]. In the respiratory network, it has been shown that 5-HT_2B_Rs enhance rhythmic motor discharge activity recorded in neonatal mice *in vitro* [35].

Until now, there were no detailed descriptions published of 5-HT_2B_R distribution in the brainstem with expression data only available for the neocortex, cerebellum, dorsal hypothalamus, and medial amygdala [36].

In the present report, we provide a detailed account of the expression and distribution of 5-HT_2B_Rs and 5-HT_2A_Rs in the ponto-medullary respiratory network including respiratory motor population of the cervical spinal cord and brainstem. Using a monospecific anti-5-HT_2B_R-antibody prepared in our laboratory and a commercially available 5-HT_2A_R antibody, we show that 5-HT_2B_Rs and 5-HT_2A_Rs are expressed in neurons of the pre-Bötzinger complex (pre-BötC), an essential kernel of the respiratory network associated with the primary rhythmogenesis [37]–[40].

Furthermore, our study also demonstrates that both receptors affect discharge properties in the phrenic motor output.

## Materials and Methods

The experimental procedures were performed in accordance with European Community and National Institutes of Health Guidelines for the Care and Use of Laboratory Animals. The Ethics Committee of the Georg-August-University, Göttingen, Germany approved the study and assigned the approval ID **T1108** to this work.

### Antibody generation

The polyclonal antibodies against the rat 5-HT_2B_R were generated by immunizing three New Zealand White rabbits (Charles River) with a 16mer peptide derived from the second intracellular loop of the rat 5-HT_2B_R amino acid sequence (NH_2_-*CAISLDRYIAIKKPIQ*-COOH; NCBI-Accession No.: NP_058946). For immunization purposes, peptides were coupled to keyhole limpet hemocyanin (KLH) according to standard protocols. The rabbits were immunized with 300 µg of KLH-coupled peptide in Hunter's adjuvant (TiterMax Gold, Sigma) five times (28-days-intervall). The resulting antiserum was affinity-purified against the immunizing peptide.

### Western Blot detection of 5-HT_2B_R protein

Brain stem tissue isolated from both male Sprague-Dawley rats and male C57BL/6J mice were resuspended in 200 µl cell lysis buffer (50 mM Tris/HCl, pH 8.0, 150 mM NaCl, 2% w/v SDS, 1% NP40, 0.5% Na-Deoxycholate) supplemented with protease inhibitor cocktail (Sigma). Protein content was determined using a Lowry assay for high SDS concentration according to manufacturer's instructions (BioRad). 40 µg of total protein of each sample were boiled in 5× Laemmli buffer (250 mM Tris/HCl, pH 6.8; 10 mM EDTA, 10% w/v SDS, 5% v/v 2-mercaptoethanol, 50% v/v glycerol, 0.5% w/v bromophenol blue) for 5 min at 95°C and then separated using a precast SDS-PAGE (Novex). Proteins were transferred to a nitrocellulose membrane. The membrane was blocked with 4% w/v BSA/TBS/0.05%Tween (pH 7.4) for 45 min at RT. 5-HT_2B_R protein was detected with a self-made monospecific polyclonal antibody (1∶1,000 dilution) after incubation for 3 hours at RT. After extensive washing, appropriate secondary horseradish peroxidase (HRP)-conjugated antibodies (Dianova, Hamburg, Germany) were used at a dilution of 1∶20,000 for 2 hours at RT. The visualization of the antigen-antibody reaction was performed with enhanced chemiluminescence (ECL) kit (BioRad, Germany).

### Immunohistochemistry

#### (a) Preparation of brain tissue

Male juvenile Sprague-Dawley rats (P25–P32) were deeply anesthetized with isoflurane (1-Chloro-2,2,2-trifluoroethyl-difluoromethylether, Abbott, Wiesbaden, Germany) until they were unresponsive to painful stimuli (pressure applied to a forepaw). A thoracotomy was performed, and animals were transcardially perfused with 50 ml of 0.9% NaCl followed by 200 ml of 4% phosphate-buffered formaldehyde (10 ml/min). The brain was removed and post-fixed for 4 hours with the same fixative at 4°C, cryoprotected in 10% sucrose for 2 hours followed by 30% sucrose in 0.1 M phosphate buffer overnight at 4°C, and then frozen at −25°C. Series of 20- and 40-µm-thick brain sections were cut from cervical spinal cord to midbrain collicular level using a freezing microtome (Frigocut, Reichert-Jung, Germany).

#### (b) Peroxidase anti-peroxidase (PAP) staining

The endogenous peroxidase activity of free-floating sections was blocked with methanol/30% H_2_O_2_ (1∶100 dilution) for 45 min at room temperature (RT) in the dark. After washing, sections were permeabilized with 0.2% Triton X-100 for 30 min. Sections were transferred for 30 min into phosphate buffered saline (PBS; pH 7.4) containing 5% bovine serum albumin (BSA) at RT for blocking non-specific binding sites. Our homemade affinity-purified rabbit anti-5-HT_2B_R antibodies or mouse anti-5-HT_2A_R antibodies (BD Bioscience, Cat. No.: 556326, San Diego, USA) were applied at a concentration of 2–5 µg/ml in a carrier-solution of 2% BSA/PBS. Sections were incubated with horseradish peroxidase (HRP)-conjugated anti-rabbit- or anti-mouse IgG antibodies (Dianova; 1∶4,000 diluted in 2% BSA/PBS) for 1 h at RT, washed, and subsequently incubated with freshly prepared diaminobenzidine (DAB) solution for 10 min at RT. Sections were mounted onto gelatine-coated microscope slides, dehydrated in increasing ethanol concentrations (2×50%, 2×80%, 2×99.9%), cleared with four changes of xylene, and finally coverslipped with DePeX (Serva, Germany).

#### (c) Immunofluorescence

Sections were permeabilized with 0.2% Triton X-100 for 30 min at RT and then washed two times with PBS (pH 7.4). Non-specific binding sites were blocked with PBS containing 5% BSA for 1 h at RT. Sections were incubated with primary antibodies (2–5 µg/ml) for 4 hours at RT. After washing, sections were incubated for 2 hours at RT in the dark with species-specific Cy2- or Cy5-conjugated secondary antibodies (Dianova, Germany; 2% BSA/PBS, antibody dilution 1∶400). Neuronal immunofluorescence was analyzed with a confocal laser-scanning microscope Meta-LSM 510 (Zeiss, Germany) using laser lines at 488 nm (Ar/Kr laser) and at 633 nm (He/Ne laser). Confocal images were processed by using overlays of two channels with the LSM 510 software provided by Zeiss. Digital images were taken at 2,048×2,048 dpi and were imported into Adobe Photoshop CS4, were digitally adjusted if necessary for brightness and contrast and were assembled into plates. Subsequent imaging procedures (cell counting) were performed using ImageJ (http://rsb.info.nih.gov/ij/).

### Molecular Biology

#### (a) Generation of expression constructs

Brain tissue from one male rat at P11 was explanted and used for total RNA isolation with the OLS RNA kit (OLS, Germany) according to manufacturer's instructions. The total RNA was used in one-step RT-PCR (Invitrogen) using primer pairs for the 5-HT_2A_R gene [*Htr2a*, F (5′-atggaaattctttgtgaagac-3′)/R (5′-tcacacacagctaaccttttc-3′)] and 5-HT_2B_R gene [*Htr2b*, F (5′-atggcttcatcttataaaatgtc-3′)/R (5′-ctatatgtagctgacttggtcttc-3′)], respectively. The cycling program used for RT-PCR comprised of: initial reverse transcription at 55°C for 30 min followed by denaturation at 94°C for 2 min. 40 cycles of denaturation at 94°C for 15 sec, annealing at 57°C for 30 sec, and elongation at 68°C for 90 sec were concluded with a final elongation step at 68°C for 5 min. The resulting RT-PCR fragment was purified from the gel and cloned into pTarget expression vector (Promega). Sequencing validated the correct insert identity.

#### (b) Transfection of cell lines

Murine neuroblastoma cell line N1E-115 was obtained from ATCC and maintained at 37°C in humid atmosphere with 5% CO_2_ and passaged every second day. For transfection, cells were seeded 24 hours prior to transfection at a density of 100,000 cells in 4-well-plates (Nunc) on acid-washed and poly-L-lysine coated 12 mm round glass cover slips. Cells were transfected with 2 µg DNA and 2 µl Lipofectamine (Invitrogen) in 500 µl OptiMEM (Invitrogen) per well and kept under normal culture conditions for 20 hours, afterwards fresh OptiMEM replaced the medium.

#### (c) Detection of endogenous Htr2b in cell lines by RT-PCR

Total RNA from 10^7^ non-transfected cells or cells transfected with 6 µg of the plasmid encoding 5-HT_2B_R was prepared using the OLS RNA kit. One µg of total RNA each was entered in the one-step-RT-PCR reaction, and the resulting PCR fragment was analyzed on an agarose gel. While the 5′-sequence of murine and rat *Htr2b* is identical, the 3′-sequences do differ. Therefore, for RT-PCR the rat forward primer was used, while the reverse primer for mouse was 5′-ctatatgtagctgacctgctcttc-3′.

#### (d) RT-PCR analyses of Htr2a and Htr2b of rat brain tissue

The total RNA of the cortex, hypoglossal nucleus, and pre-BötC dissected from corresponding 300-µm-thick slice preparations was isolated using GenElute™ mammalian total RNA kit (Sigma). First strand cDNA was synthesized from 1 µg total RNA using SuperScript™ first-strand synthesis system with random hexamers according to manufacturer's instructions (Invitrogen). Samples without reverse transcription (w/o RT) served as negative controls for the following PCR to exclude amplification of genomic DNA. For the PCR, specific forward and reverse primers were derived from different exons of the 5-HT_2A_R and 5-HT_2B_R cDNA to avoid amplification of genomic DNA. The cDNA sequences were obtained from the National Center for Biotechnology Information (NCBI; http://www.ncbi.nlm.nih.gov/). Specificity of selected primers was tested by partial sequencing of the amplification products for their identification by SeqLab company (Göttingen, Germany). The following primer pairs were used for amplification:

5-HT_2A_R [*Htr2a*, NCBI-Accession No.: NC_005114.2; F (5′-accttgtgtgtgagtgacct-3′)/R (5′-taggccaatgctggtatagt-3′)], 5-HT_2B_R [*Htr2b*, NC_005108.2; F (5′-ctggttattctggctgtttc-3′)/R (5′-gaccacatcagcctctattc-3′)], and for β-actin [*Actb*, NC_005111.2; F (5′-gatatcgctgcgctcgtcgtc-3′)/R (5′-cctcggggcatcggaacc-3′)].

The PCR reaction mixture for one sample was composed of 1–2 µl cDNA, 1 µl forward primer, 1 µl reverse primer, 1 µl dNTPs (100 mM dNTP mix), 1 µl DMSO, 5 µl NH_4_ buffer (10×), 2 µl MgCl_2_ (50 mM solution), and 1 µl PANScript red DNA polymerase (PAN Biotech, Germany). The mixture was filled up to 50 µl with DEPC-treated water. The following program was used for the PCR reaction: *initial denaturation* at 94°C, 4 min/38×[*denaturation* 94°C, *annealing* 1 min/55°C, *extension* 1 min/72°C, 2 min]/*final elongation* 72°C, *termination* 10 min/4°C hold. *Actb* (β-Actin) was used as an internal standard for all PCR reactions.

#### (e) Real-time RT-PCR

The relative quantification of *Htr2a* and *Htr2b* gene expression in specific rat tissues was done by real-time RT-PCR analysis. Spinal cord, inferior olive, pre-Bötzinger complex, and parabrachial complex were dissected from corresponding 300-µm-thick cryostat sections (P32; n = 3 animals) under visual control. The total ribonucleic acid (RNA) of homogenized brain tissue was isolated using the Trizol® method according to manufacturer's instructions (GibcoBRL) and its concentration was determined using the NanoDrop ND-1000 spectrophotometer followed by its quality and integrity measurement by electrophoresis on RNA 6000 LabChip® kit (Agilent 2100 Bioanalyzer). The RNA was transcribed into the corresponding deoxyribonucleic acid (cDNA) using the iScript cDNA Synthesis Kit (BioRad). The following primer pairs were designed by using the Primer3 program (http://frodo.wi.mit.edu/primer3/):

*Htr2a* (NM_017254.1): F (5′-tgtcgccatccagaacccca-3′)/R (5′-gcaggcagctcccctcctta-3′); *Htr2b* (NM_017250.1): F (5′-agactgccgagaaccagggg-3′)/R (5′-gcggtggctgatttgctggt-3′); *Hprt1* (NM_012583.2): F (5′-gtcaagcagtacagccccaaaatg-3′)/R (5′-gtttgttgttggatatgcccttgac-3′).

Gel electrophoresis revealed a single polymerase chain reaction (PCR) product, and the melting curve analysis showed a single peak for all amplification products. The PCR products were sequenced and blasted to confirm the correct identity of each amplicon. Ten-fold serial dilutions generated from cDNA of each sample were used as a reference for the standard curve calculation to determine primer efficiency. Triplicates of all real-time PCR reactions were performed in a 25 µl mixture containing 1/20 volume of the sample cDNA preparation from 250 ng total RNA, 400 nM of each primers, and 1× Fast-SYBR Green Master Mix (Applied Biosystems, USA).

The PCR-reactions were performed as follows: *initial activation* at 95°C for 60 s, 42 cycles of (*denaturation* 95°C/10 s, *annealing* and *extension* 60°C/30 s), and a final gradual increase of 0.5°C in temperature from 60°C to 90°C.

All real-time quantifications were performed using the iCycler iQ system (BioRad) and were adjusted by using the method according to Pfaffl [41].

### Calcium imaging of cells recombinantly expressing 5-HT_2A_Rs or 5-HT_2B_Rs

The perfused brainstem preparation is, due to its thickness and need for constant perfusion not suited for microscopic analysis. Therefore, we opted to do the calcium imaging in murine neuroblastoma N1E-115 cells, where endogenous expression of 5-HT_2_Rs is negligible, but are known to signal via the PLC-DAG pathway [42], [43]. Another advantage of transfection is the control over which receptors (5-HT_2A_R, 5-HT_2B_R or both) are expressed in individual cells, avoiding the need for antagonists and simplifying analysis. 12–16 hours post transfection, cells were transferred to calcium-free imaging medium (130 mM NaCl, 3.5 mM KCl, 1.25 mM NaH_2_PO_4_, 24 mM NaHCO_3_, 1.2 mM MgSO_4_, 10 mM Glucose) and incubated with Fluo-4-AM (Invitrogen) at a final concentration of 5 µM for 30 min at 37°C. The Fluo-4-AM stock solution was prepared as 2 mM using 10% pluronic acid F-127 in DMSO (Sigma) and was diluted just before use. After incubation, cells were washed with calcium-free medium and Fluo-4-AM was allowed to hydrolyze for another 30 min in the presence of probenicid to avoid leeching of fluorescent probe from the cell.

For calcium imaging, Fluo-4-AM loaded cells were transferred to a recording chamber equipped with an inverted Olympus microscope (IX71) with appropriate filters (515 nm beam splitter and a 535/50 band-pass filter) and a triggered LED light source (PreciseExcite, CoolLED) with 465 nm excitation. Images were taken for 300 µsec at 1 sec intervals. After recording baseline fluorescence, cells were stimulated with 1000 nM serotonin (Sigma) in calcium-free medium. This concentration was chosen based on a dose-response curve giving a linear response for serotonin stimulation between 500 and 1500 nM. For all experiments, a 10×, 1.0 NA objective (Olympus) was used.

To compare calcium measurements between experiments, we calculated the apparent fluorescent intensity F/F_0_ by dividing the fluorescent intensity (F) at every time point by the average fluorescence recorded before stimulation (F_0_). Data were statistically analyzed with Student's t-test and presented as mean ± standard deviation (s. d.).

### Perfused brainstem preparation of rats

#### (a) Perfused brainstem preparation of rats

The experiments on the perfused brainstem preparation [44] were performed on male Sprague-Dawley rats (P25–P32, 90–150 g) that were housed under a 12 h light/dark cycle, with food and water provided ad libitum.

Animals were deeply anesthetized with halothane until they were unresponsive to a forepaw pinch, decerebrated at the pre-collicular level and cerebellectomized, bisected below the diaphragm, and the skin was removed. The upper body was placed into a recording chamber and perfused retrogradely via the thoracic aorta with ACSF (containing in mM: MgSO_4_ 1.25; KH_2_PO_4_ 1.25; KCl 5; NaCl 125; CaCl_2_ 2.5; NaHCO_3_ 25; glucose 10, 1.25% Ficoll and aerated with carbogen (5% CO_2_/95% O_2_; pH 7.35 at 30°C). The perfusate was collected, filtered twice and re-circulated. Norcuronium-bromide (0.5 mg/200 ml) was added for muscle relaxation. The perfusion pressure was set to 45 to 55 mm Hg.

#### (b) Phrenic nerve signal processing

A silver wire immersed in bath solution within a capillary suction electrode picked up phrenic nerve activity representing the respiratory motor output to the diaphragm and inspiratory rib cage muscles. Phrenic nerve signals were amplified 2,000–5,000×, filtered (low-pass, 7,000 Hz cutoff frequency; high pass, 8 Hz) and integrated (time constant, 100 ms). The processed signals were digitized by a PowerLab 8/30 microprocessor and stored using LabChart 7 software (ADInstruments, Australia).

Drugs added to the perfusate for specific pharmacological manipulation of 5-HT_2_Rs were purchased from Tocris Bioscience, Ellisville, USA: 5-HT_2A_R agonist TCB-2 and 5-HT_2A_R antagonist Altanserin hydrochloride, 5-HT_2B_R agonist BW 723C86 and 5-HT_2B_R antagonist LY 272015.

#### (c) Analysis of phrenic nerve discharge properties

Discharges of a representative one-minute duration were measured in the absence of (control) and after intra-arterial perfusion with ACSF containing a 5-HT_2A_ or 5-HT_2B_ receptor agonist or antagonist. Measurements of drug effect were made at 5-minute intervals. The peak of the integrated discharge (mV) was used as an estimator of discharge intensity and normalized to the control, which was set to 100%. Discharge frequency (bursts per minute) was calculated from the integrated signals. Values (mean ± standard error of the mean) for amplitude and frequency were calculated from consecutive discharges that occurred over one minute during control and when drug effects were maximal.

All statistical tests (paired t-test) for pharmacological experiments were performed using GraphPad Prism version 5.0d for MacOS X.

### Test drugs

5-HT receptor ligands tested for effects on phrenic nerve discharge properties were purchased from Tocris Bioscience, USA: TCB-2 (5-HT_2A_R agonist), Altanserin hydrochloride (5-HT_2A_R antagonist), BW 723C86 (5-HT_2B_R agonist), LY 272015 (5-HT_2B_R antagonist).

## Results

### Production and characterization of monospecific anti-5-HT_2B_R antibodies

**Figure 1:**
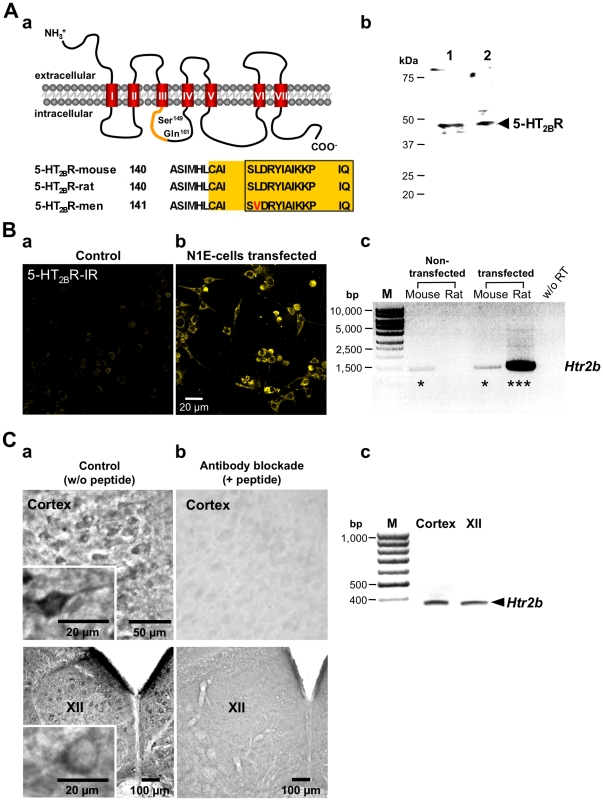
Verification of the anti-5-HT_2B_R antibody. (**A**) (**a**) The 5-HT_2B_R belongs to the family of seven-transmembrane-domain receptors that are coupled to hetero-trimeric guanine-nucleotide-binding protein q (G_q_). The transmembrane-domains are indicated by red cylinders (I–VII). We produced a novel monospecific polyclonal antibody against the 5-HT_2B_R by selecting a specific amino acid sequence (Cys^146^ - Gln^161^; NH_2_-*CAISLDRYIAIKKPIQ*-COOH) of the second intracellular loop of the rat 5-HT_2B_R-sequence. The peptide exhibits 100% homology in mouse. Red letters indicate mismatches in the human-sequence (L→V). (**b**) Immunoblot analysis of mouse (**1**) or rat (**2**) brainstem lysate revealed a specific band at about 48 kDa that corresponds with the predicted relative molecular mass of the 5-HT_2B_R. (**B**) 5-HT_2B_R expression in non-transfected (**a**) and transfected N1E-115 cells (**b**). The anti-5-HT_2B_R antibody-dependent staining indicated a strong labeling of N1E-115 cells that had been transiently transfected with the rat 5-HT_2B_R (**b**). Non-transfected cells expressing the mouse 5-HT_2B_R showed a weak neuronal immunofluorescent signal that corresponds with a weak PCR signal (amplicon size 1114 bp) for the mouse 5-HT_2B_R-mRNA (*Htr2b*) (**c**). Samples without reverse transcription (w/o RT) served as negative controls. (**C**) (**a**, **b**) Immunohistochemistry. Both pyramidal neurons of the cortex and motoneurons of the hypoglossal nucleus (XII) revealed a strong 5-HT_2B_R immunoreactivity (-IR) (**a**) that was effectively blocked after pre-incubation of the antibody with a 50-fold molar excess of the peptide *CAISLDRYIAIKKPIQ* (+ peptide) that was used for immunization (**b**). Insets in (**a**) show labeled neurons at a higher magnification. Immunolabeling was performed using the PAP-method with diaminobenzidine as chromogen. (**c**) RT-PCR analysis of the rat cortex and hypoglossal nucleus. The 5-HT_2B_R-specific mRNA (*Htr2b*) was detectable in neurons within both the rat cortex and the hypoglossal nucleus (XII) (amplicon size 380 bp).

The peptide for immunization was derived from the second intracellular loop of the rat 5-HT_2B_R-sequence (NH_2_-*CAISLDRYIAIKKPIQ*-COOH; fig. 1Aa). The specificity of the monospecific polyclonal anti-5-HT_2B_R antibody was tested in three different test systems: Immunoblot analysis (n = 3) of both mouse and rat brainstem lysate revealed a specific band at 48 kDa, which is in accordance with the predicted relative molecular mass of the receptor (fig. 1Ab). Murine neuroblastoma cells recombinantly expressing rat 5-HT_2B_R (fig. 1B) were used for specificity testing of the antibody, while the neocortex and the hypoglossal nucleus (XII) was selected to test immunohistochemistry in tissue (fig. 1C) based on previous positive results reported by Duxon [36].

The control cells faintly expressed the mouse 5-HT_2B_R that is also recognized by the antibody because of sequence homology. After transfection with the rat receptor the antibody labeling revealed a strong fluorescent signal. Also, both brain regions selected showed strong 5-HT_2B_R reactivity (fig. 1Bc).

The anti-5-HT_2B_R antibody immunoreactivity on cells as well as on neurons of both regions was effectively blocked after pre-incubation of the primary antibody with a 50-fold molar excess of the peptide that was used for immunization indicating specificity. As a control, RT-PCR analysis confirmed 5-HT_2B_R-specific mRNA expression in cells within both regions (fig. 1Cc).

### Expression analysis of 5-HT_2A_ and 5-HT_2B_Rs in the respiratory network

**Figure 2:**
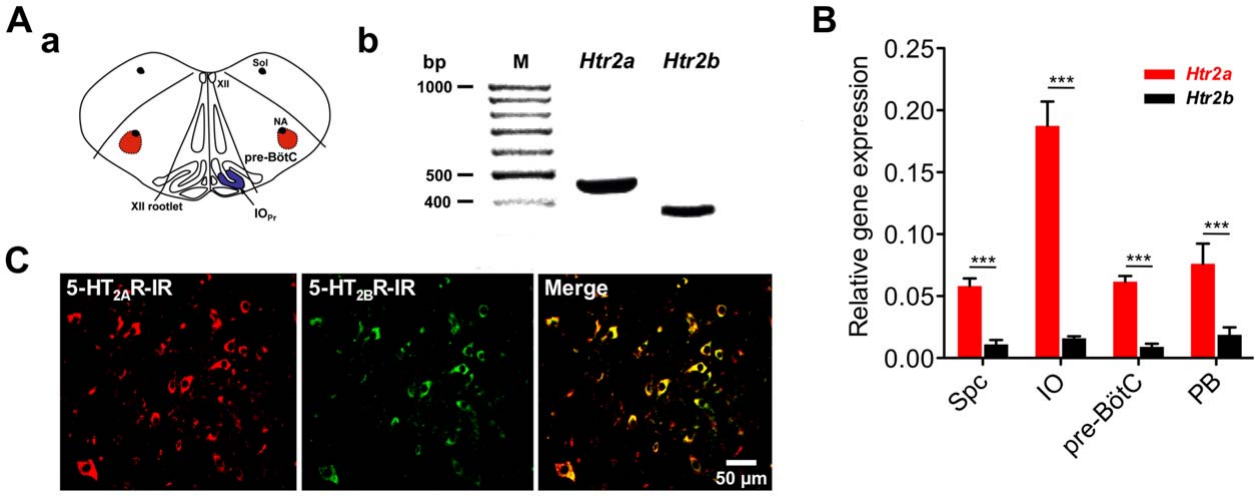
Quantification of expression levels and co-expression of 5-HT_2A_ and 5-HT_2B_Rs within the pontine respiratory network. (**A**) (**a**) shows a schematic representation of the dissected pre-BötC and its landmarks: pre-Bötzinger complex (pre-BötC), nucleus of the solitary tract (Sol), nucleus ambiguus (NA), hypoglossal nucleus (XII), principal nucleus of the inferior olive (IO_Pr_). (**b**) shows the specific mRNA of both receptors detected in the pre-BötC. (**B**) Double labeling of 5-HT_2A_ and 5-HT_2B_Rs. 5-HT_2A_ (Cy5, red) and 5-HT_2B_Rs (Cy2, green) are strongly co-expressed in pre-BötC-neurons. Immunohistochemical analysis does not reveal the ratio of co-expressed proteins. Therefore, we performed quantitative real-time RT-PCR on four selected nuclei of the respiratory network (**C**). The bar diagram represents results of quantitative real-time RT-PCR analysis of 5-HT_2_R genes (*Htr2a*, *Htr2b*) of spinal cord (Spc), inferior olive (IO), pre-Bötzinger complex (pre-BötC), and parabrachial complex (PB). At the RNA level 5-HT_2A_R is significantly stronger expressed compared to *Htr2b* in all regions analyzed. Asterisks indicate significance (*** = p<0.001; ANOVA with Bonferroni's post hoc test).

Prior to analysis of 5-HT_2A_R and 5-HT_2B_R expression at the protein level, we confirmed their expression at the RNA level. For this, we dissected specific brain stem regions (fig. 2Aa) from corresponding frozen cryostat sections of juvenile rats (P30; n = 3 in which patches of both sides of each section were combined for one sample). Conventional RT-PCR analysis revealed gene expression for 5-HT_2A_ (*Htr2a*) and 5-HT_2B_R (*Htr2b*) in the pre-BötC at the RNA level (fig. 2Ab). However, relative quantification of gene expression using real-time RT-PCR analysis revealed a 5.3-fold stronger expression of the *Htr2a* compared to the *Htr2b* gene in the spinal cord (0.0582±0.0061 vs. 0.0110±0.0036), 11.8-fold in the inferior olive (0.1875±0.0196 vs. 0.0159±0.0016), 6.8-fold in the pre-BötC (0.0617±0.0046 vs. 0.0091±0.0024), and a 4.1-fold stronger one in the parabrachial complex (0.0671±0.0162 vs. 0.0188±0.0061) (fig. 2C).

**Figure 3:**
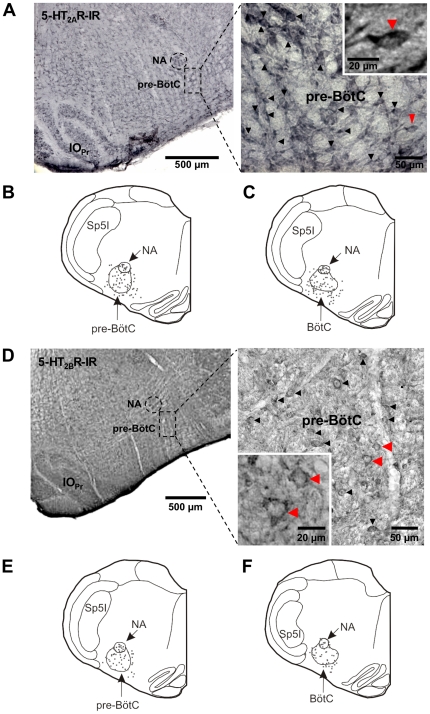
Expression patterns of 5-HT_2A_ and 5-HT_2B_Rs within the medullary respiratory network. (**A**) 5-HT_2A_R expression pattern in the pre-BötC. The plots (**B**, **C)** represent 5-HT_2A_R immunoreactivity within the pre-BötC (**B**) and BötC (**C**). (**D**–**F**) shows the corresponding expression pattern for the 5-HT_2B_R. The insets show labeled neurons at a higher magnification. Abbreviations: Bötzinger complex (BötC), nucleus ambiguus (NA), pre-Bötzinger complex (pre-BötC), principal nucleus of the inferior olive (IO_Pr_), interpolar spinal trigeminal nucleus (Sp5l).

**Figure 4:**
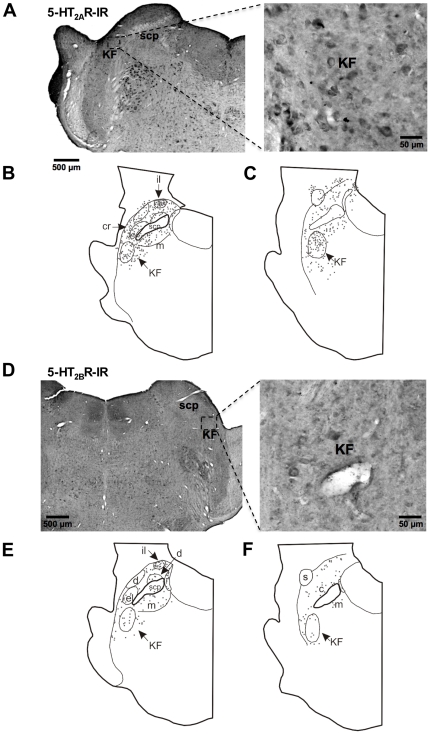
Expression patterns of 5-HT_2A_ and 5-HT_2B_Rs within the pontine respiratory network. Both 5-HT_2A_ and 5-HT_2B_ receptors are abundantly expressed in neurons of the internal lateral nucleus of the parabrachial complex (**B**, **E**). Within the nucleus Kölliker-Fuse the 5-HT_2B_R expression is weak compared to 5-HT_2A_Rs (**A**, **D**). Abbreviations: internal lateral nucleus of the PB (il), lateral crescent nucleus of the PB (cr), nucleus Kölliker-Fuse (KF), parabrachial complex (PB), superior cerebellar peduncle (scp).

To analyze receptor expression at the protein level within the ponto-medullary respiratory network we applied our self-made monospecific polyclonal anti-5-HT_2B_R antibody in combination with a commercially available monoclonal anti-5-HT_2A_R antibody (BD Bioscience, San Diego, USA). Both receptor subtypes were expressed in crucial parts of the respiratory network such as the pre-BötC and the pontine Kölliker-Fuse nucleus (fig. 3, 4). A detailed analysis of the pre-BötC, the supposed kernel essential for the generation of the primary respiratory rhythm (Smith et al., 1991), showed a strong co-expression of 5-HT_2A_R and 5-HT_2B_R.

We analyzed a total amount of 2136 5-HT_2A_R-immunoreactive cells (5 sections from each animal, n = 5 animals). 1345 cells (63%) of these cells also expressed the 5-HT_2B_R indicating strong co-expression of 5-HT_2A_R and 5-HT_2B_R (fig. 3).

In the dorsolateral pons expression of 5-HT_2B_R within the parabrachial complex (PB) and Kölliker-Fuse (KF) nucleus was weak, compared to the 5-HT_2A_R (fig. 4D–F). Contrary, the 5-HT_2A_R showed dense expression in the KF and lateral crescent nucleus of the PB (fig. 4A–C). Both nuclei are closely linked with respiratory control. In addition more modest expression was observed in the external lateral, central, and dorsal nuclei of the PB. Curiously, both 5-HT_2A_R and 5-HT_2B_R showed dense expression in the internal lateral nucleus (il) of the PB (fig. 4B, E), a subnucleus of the PB-complex that is still undefined in its physiological functions.

### Analysis of systemic effects of 5-HT_2A_Rs and 5-HT_2B_Rs on respiratory activity

The effects of systemic application of specific antagonists for 5-HT_2A_R and 5-HT_2B_R (Altanserin hydrochloride and LY 272015, respectively) and specific agonists for 5-HT_2A_R and 5-HT_2B_R (TCB-2 and BW 723C86, respectively) on spontaneous breathing activity were investigated in male Sprague-Dawley rats using the perfused brainstem preparation [44]. We found that pharmacological manipulation of 5-HT_2_Rs can either change the amplitude or the frequency of the phrenic nerve activity (PNA).

**Figure 5:**
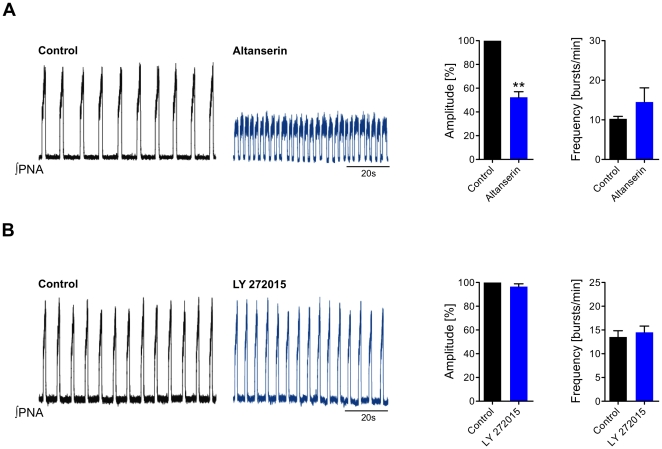
Respiratory network responses to systemic 5-HT_2A_R and 5-HT_2B_R antagonist applications of in situ rats. Shown are representative traces of integrated phrenic nerve activity (∫PNA) of 2 different experimental conditions. Black traces represent the ∫PNA under control conditions and blue traces indicate application of antagonists. The bar diagrams on the right give the averages of amplitude and frequency of least 3 independent experiments of each condition. Statistical analysis (paired t-test) is denoted in the panels on the right side with asterisks indicating significance (** = p<0.01). (**A**, **B**) Action of 5-HT_2A_R and 5-HT_2B_R antagonists in the perfused brainstem preparation of rat. (**A**) Application of the specific 5-HT_2A_R antagonist Altanserin (blue trace) significantly decreased phrenic nerve activity (PNA) by decreasing the amplitude. Also, a slight increase in frequency was observed. (**B**) Application of the 5-HT_2B_R antagonist LY 272015 had no discernable effect on either amplitude or frequency of ∫PNA. We interpret these findings as constitutive activity of 5-HT_2A_R.

**Figure 6:**
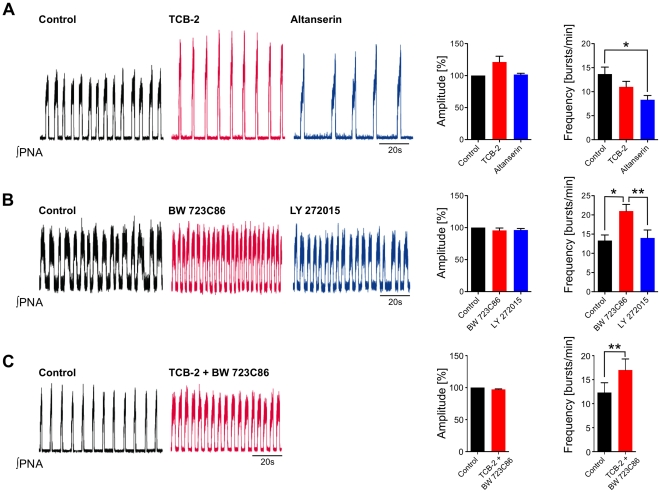
Respiratory network responses to systemic 5-HT_2A_R and 5-HT_2B_R activation. Shown are representative traces of integrated phrenic nerve activity (∫PNA) of 3 different experimental conditions. Black traces represent the ∫PNA under control conditions, while red traces indicate application of agonists and blue traces indicate application of antagonists. The bar diagrams on the right give the averages of amplitude and frequency of least 3 independent experiments of each condition. Statistical analysis (paired t-test) is denoted in the panels on the right side. (**A**–**C**) Action of 5-HT_2A_R and 5-HT_2B_R ligands in the perfused brainstem preparation. (**A**) Application of the specific 5-HT_2A_R agonist TCB-2 (red trace) increased phrenic nerve activity (PNA) by increase of the amplitude, and subsequent application of the specific 5-HT_2A_R-antagonist Altanserin (blue trace) reduced the amplitude. PNA frequency decreased below control. (**B**) Application of the 5-HT_2B_R agonist BW 723C86 increased PNA frequency. After subsequent administration of the specific 5-HT_2B_R-antagonist LY 272015 phrenic nerve activity returned nearly to baseline level. (**C**) Simultaneous application of the specific agonists TCB-2 and BW 723C86 only increased the frequency.

Application of the 5-HT_2A_R-antagonist Altanserin hydrochloride (8.0 µg/ml, n = 3) [32], [45] significantly reduced the amplitude from control of 100% to 52.34±4.72% (p<0.01), while breathing frequency increased from control of 10.25±0.63 to 14.50±3.59 bursts/min; n.s. (fig. 5A). Blockade of 5-HT_2B_R with the antagonist LY 272015 (2.87 µg/ml, n = 3) [35], [46] did not significantly changed both the amplitude (100% of control vs. 96.54±2.36%; n.s.) and frequency (13.50±1.32 bursts/min of control vs. 14.50±1.32 bursts/min; n.s.) (fig. 5B). Application of the 5-HT_2A_R-agonist TCB-2 (0.67 ng/ml, n = 5) [47] caused an increase of the amplitude (from 100% of control to 121.3±8.99; n.s.), while respiratory frequency slightly decreased (13.67±1.45 bursts/min of control vs. 11.00±1.12 bursts/min; n.s.). Administration of the 5-HT_2A_R-antagonist Altanserin caused a further decrease of frequency to 8.33±0.88; p<0.05; (fig. 6A). Application of the 5-HT_2B_R-agonist BW 723C86 (0.67 µg/ml, n = 5) [32] only caused an increase in frequency (13.33±1.45 bursts/min of control vs. 21.00±1.73 bursts/min; p<0.05), while the amplitude was not affected (from 100% of control to 95.67±3.76%; n.s.). Blockade of the 5-HT_2B_R using LY 272015 diminished respiratory frequency nearly to baseline level (14.00±2.08 bursts/min; p<0.01) (fig. 6B). Interestingly, the simultaneous application of both agonists together resulted only in an increase of frequency (from 12.33±2.03 to 17.00±2.31 bursts/min; p<0.01), whereas no increment of amplitude could be observed (100% of control vs. 97.33±0.67%; n.s.; fig. 6C).

### Calcium imaging of cells recombinantly expressing 5-HT_2A_R or 5-HT_2B_R

**Figure 7:**
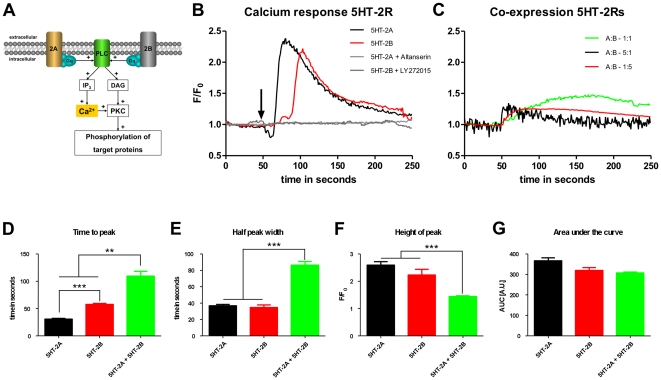
Signal transduction pathways of 5-HT_2A_ and 5-HT_2B_ receptors and calcium imaging. (**A**) The schematic diagram illustrates convergent signaling pathways for 5-HT_2_Rs. (**B**) shows summed calcium transients recorded in N1E-115 cells transfected with either 5-HT_2A_R (red line) or 5-HT_2B_R (black line). The curves were constructed from the means of 3–5 independent experiments, averaging at least 30 individual measurements. The arrow indicates the time of stimulation. Light and dark grey curves indicate control experiments of 5-HT_2A_R stimulated with Altanserin and 5-HT_2B_R stimulated with LY272015, respectively. (**C**) shows summed calcium transients recorded in N1E-115 cells transfected with a stiochiometric ratio of 5-HT_2A_R∶5-HT_2B_R of 1∶1 (green line), 5-HT_2A_R∶5-HT_2B_R of 5∶1 (black line) and 5-HT_2A_R∶5-HT_2B_R of 1∶5 (red line). The bar diagrams (D–G) show statistical analysis of (**D**) height of peak (F/F_0_), (**E**) time to peak (s), (**F**) half peak width (s) and (**G**) area under the curve (AU) for expression of 5-HT_2A_R alone (black), 5-HT_2B_R alone (red) or expression of 5-HT_2A_R and 5-HT_2B_R together at a stiochiometric ratio of 1∶1 (green). Asterisks indicate significance (*** = p<0.001; ** = p<0.01; paired t-test). Abbreviations:, serotonin 2A receptor (2A), serotonin 2B receptor (2B), phospholipase C (PLC), inositol 1, 4, 5-triphosphate (IP_3_), diacylglycerol (DAG), protein kinase C (PKC), hetero-trimeric guanine-nucleotide-binding protein q (G_q_), adenylyl cyclase (AC), cyclic adenosine 5′, 3′-monophosphate (cAMP), protein kinase A (PKA).

The electrophysiological recordings showed a frequency increase controlled by 5-HT_2B_Rs when both receptors were stimulated concomitantly (fig. 5C), although both 5-HT_2A_R and 5-HT_2B_R are coupled to the same G_q_-mediated signaling pathway (fig. 7A).

To analyze the calcium signaling of both receptors, we expressed them recombinantly either alone or together in neuroblastoma cells. To avoid artifacts, relatively low amounts of DNA were transfected to avoid overexpression.

The recombinant approach allowed us to image single cells and correlate resulting signals to a defined complement of receptors, which would have not been possible in perfused brainstem preparations.

In neuroblastoma cells expressing 5-HT_2A_Rs and 5-HT_2B_Rs alone, either agonist evoked a release of cytosolic Ca^2+^ from intracellular stores (see figure 7 and table 1) with a large initial calcium spike. While the reactions of individual cells varied slightly, the mean Ca^2+^ increase of cells expressing 5-HT_2A_R (peak F/F_0_ of 2.59±0.8) was similar to those expressing 5-HT_2B_R (peak F/F_0_ of 2.23±1.1). In contrast, the calcium increase was significantly faster for 5-HT_2A_R (time to peak 30.5±9.6) than for 5-HT_2B_R (time to peak 57.7±8.4). The calcium level almost returned to baseline levels within ∼35 seconds exhibiting no significant differences for both receptors, with a half-peak-width of 36.7±1.7 sec for 5-HT_2A_R and 34.6±3.2 for 5-HT_2B_R (fig. 7B).

**Table 1:** Effects of 5-HT_2A_R and 5-HT_2B_R agonists on Ca^2+^ fluorescence signals.

Ca^2+^ Signal Properties	5-HT_2A_R-agonist	5-HT_2B_R agonist	both agonists	Unit	T-test*	T-test**
Onset time	10.1±1,7	40.3±1.5	11.9±2.3	[sec]	p, 0.001	p, 0.01^a^
Time to peak	30. 5±9.6	57.5±8.4	79.44±8.9	[sec]	p, 0.01	p, 0.01
Half peak width	36.7±1.7	34.6±3.2	86.32±5.0	[sec]	p, 0.500	p, 0.001
Height of peak (F/F_0_)	2.59±0.8	2.23±1.1	1.44±0.17	[AU]	p, 0.500	p, 0.001
Area under the curve	366.38±93.64	319.4±74.86	307.43±24.43	[AU]	p, 0.500	p, 0.500

* comparing results from single transfected cells stimulated with 5-HT_2A_R-agonist or with 5-HT_2B_R agonist.

** comparing results from both single transfected/stimulated cells against double transfected/double stimulated cells.

a only when compared to single transfected cells stimulated with 5-HT_2A_R.

Co-application of 5-HT_2A_R and 5-HT_2B_R agonists had unexpected effects. The Ca^2+^ signal (79.44±8.9 sec) was significantly slower from onset to peak than the signals produced by either agonist alone (p<0.001), while the time of onset was similar to 5-HT_2A_R alone. In addition, the fluorescence signal at its peak (F/F_0_, 1.44±0.17) was notably smaller (p<0.001). While the duration of the calcium peak by co-stimulation of both receptors was nearly doubled (half-peak-width of 86.32±5.0 sec; p<0.001), the amount of released calcium, measured as “area under the curve”, was very similar no matter if the receptors are expressed alone or together.

As our real-time PCR analysis showed that 5-HT_2A_R and 5-HT_2B_R are expressed in different amounts, with 5-HT_2A_R being in 5- to 10-fold excess, we also transfected N1E cells with DNA ratios of 5-HT_2A_R to 5-HT_2B_R of 5∶1 and 1∶5, respectively. Regardless of the DNA ratios, the presence of 5-HT_2B_R always produced slow but wide calcium transients (as shown for 5-HT_2A_R and 5-HT_2B_R in fig. 7C).

## Discussion

This study reveals the locations of 5-HT_2A_R and 5-HT_2B_R in regions of the ponto-medullary respiratory network, including sites where the receptors are co-expressed. We demonstrate that agonist activation of the receptors evokes dramatic changes in discharge activity recorded from the phrenic motor output, and that activation of each type of receptor has distinctive effects on phrenic nerve discharge intensity and duration. Through the use of selective receptor antagonists, we found that only the 5-HT_2A_R constitutively modulates phrenic motor output. In neuroblastoma cells transfected with 5-HT_2A_R and 5-HT_2B_R, we discovered distinctly different calcium signal kinetics when each type of 5-HT receptor was activated. We also uncovered unexpected effects on signal amplitude and time course when 5-HT_2A_R and 5-HT_2B_R are coactivated.

In the paragraphs to follow, we discuss each of these aspects in turn, along with their physiological implications for respiratory motor output modulation.

### Distribution of 5-HT_2B_ and 5-HT_2A_ receptors in regions of the ponto-medullary respiratory network

Within the medulla and pons, functionally defined respiratory regions provide input to cranial motoneurons controlling the airways, and to spinal motoneurons activating inspiratory and expiratory pump muscles. A variety of neurotransmitters and modulators involved in respiratory control have been identified in many of these respiratory related compartments (reviewed by Alheid and McCrimmon [48]). In all these regions, 5-HT_2A_R and coupled protein kinase dependent signaling pathways have been identified functionally and anatomically [49]. Until now, however, the distribution of 5-HT_2B_Rs had not been investigated, and nothing had been known about their functional importance to respiratory control. Our study shows that 5-HT_2A_R and 5-HT_2B_R are co-localized in the Kolliker-Fuse and Parabrachial regions of the Pons, and in the BötC and pre-BötC of the ventral medulla with an approximate 5-fold stronger expression of 5-HT_2A_R in all regions.

Respiratory neurons in the PB and KF constitute the pontine respiratory group. A variety of respiratory neuronal types are found in this region [50], [51], which receives axonal projections from the ventral respiratory column, and from the nucleus of the solitary tract (NTS). The NTS itself is a receiving station for pulmonary afferents from the lungs and upper airways. Based on 5-HT_2A_R and 5-HT_2B_ R co-expression patterns, our study would predict contributions by both types of receptors to respiratory modulation within the KF-PB complex, with a modulatory role played by 5-HT_2B_Rs.

Based on both *in vitro* and *in vivo* [52], [53] studies, the pre-BötC was identified as a medullary region essential for respiratory rhythm generation. The region may play a prominent role in inspiratory phase control, although the pre-BötC contains populations of neurons that exhibit a variety of respiratory related discharge patterns (Schwarzacher et al., 1995). Previously, 5-HT_1A_, 5-HT_2A_, 5-HT_4_, and 5-HT_7_ receptors have been identified in the pre-BötC by immuno-labeling [8], [12], [17]. The BötC houses a prominent population of expiratory neurons that provide widespread inhibitory projections within the ventral respiratory column (VRC), targeting both inspiratory and expiratory bulbospinal neurons as well as respiratory-related cranial motoneurons [54]. Some expiratory BötC neurons also send axon collaterals to the spinal cord, reaching at least as far as the phrenic motor nucleus [48], [55]. Our present study of 5-HT_2A_R and 5-HT_2B_R co-expression in the pre-BötC and BötC suggests that, as in the pontine respiratory group, 5-HT_2A_R modulation is predominant.

### Differential effects of 5-HT_2A_ and 5-HT_2B_ receptor agonists and antagonists on phrenic nerve discharge properties

Activation of 5-HT_2A_Rs by the selective, CNS-permeable agonist TCB-2 [47], [56] increased PNA discharge amplitude but not frequency, whereas the 5-HT_2A_R antagonist Altanserin decreased both amplitude and frequency: in fact, discharge frequency decreased below control level. Although speculative, we suggest that 5-HT_2A_R agonists target two functionally different populations of respiratory neurons: bulbospinal inspiratory neurons, leading to increased phrenic motor output, and propriobulbar inspiratory phase-regulating neurons that determine discharge frequency. Altanserin's capacity to reduce discharge frequency below control levels indicates that constitutive 5-HT_2A_R activation is substantial in inspiratory phase regulating neurons.

The 5-HT_2B_R agonist BW 723C68 increased only frequency, indicating that only neurons involved in discharge rate regulation were affected. However, the antagonist LY 272015 changed neither amplitude nor frequency. This suggests that in our *in situ* perfused brainstem preparation, 5-HT_2B_Rs though present and activated by BW 723C68 were not constitutively activated.

When 5-HT_2A_R and 5-HT_2B_R agonists were given concurrently, a frequency increase that exceeded the singular effects of either agonist occurred. This is an expected outcome if frequency-controlling neurons are preferred targets for 5-HT_2B_R modulation in the ponto-medullary respiratory compartment.

### 5-HT_2B_ and 5-HT_2A_ receptors effects on Ca^2+^ signaling

Because 5-HT_2A_ and 5-HT_2B_ receptors both utilize an intracellular signal pathway that leads to a buildup of cytoplasmic Ca^2+^, we used the Ca^2+^ signal as a measure of receptor activation by TCB-2 and by BW 723C68. Neuroblastoma cells, for reasons presented earlier, are advantageous for measuring the magnitude and kinetics of intracellular Ca^2+^ fluctuations. Nonetheless, we acknowledge that there are limitations in relating Ca^2+^ signals detected in neuroblastoma cells to discharge properties recorded from the phrenic motor output, and we interpret our results with this caveat in mind.

Agonist activation of 5-HT_2A_ receptors produced a Ca^2+^ signal that was rapid in onset, somewhat faster in time course to peak and larger in magnitude than the Ca^2+^ transient produced by 5-HT_2B_ receptor activation. Another characteristic difference was an initial small dip in the signal due to 5-HT_2A_ receptors, whereas a small Ca^2+^ transient preceded the predominant 5-HT_2B_R dependent signal. Their respective signal profiles may reflect differences in dynamic interactions involving receptor activation, cell membrane Ca-channel openings as well as Ca^2+^ release and uptake processes in intracellular organelles. A detailed interpretation of Ca^2+^ signals recorded from the respiratory rhythmic rodent slice preparation can be found in published studies by Keller and coworkers [57], [58]. Those studies illustrate Ca^2+^ signals that are proportional to respiratory discharge intensity. Assuming that Ca^2+^ signaling in neuroblastoma cells can be equated with signaling in cells of the brainstem respiratory network, we interpret our findings as follows. The faster and larger Ca^2+^ signal may reflect more efficient 5-HT_2A_R coupling to the PLC-DAG-PKC signal pathway, or to the spatial arrangement of receptor, Ca^2+^-channel, and organelles involved in Ca^2+^ release and uptake. We can offer no ready explanation for the slowing and diminution of the Ca^2+^ signal when 5-HT_2A_ and 5-HT_2B_ receptors co-expressed in neuroblastoma cells were coactivated. Perhaps different isoforms of DAG or PKC activated by 5-HT_2A_R and 5-HT_2B_R compete for phosphorylation sites on membrane calcium channels and storage sites and interact negatively.

### Conclusion

Taking together the results from all sets of experiments (distribution, co-expression, phrenic nerve activity, and Ca^2+^ signaling), we formulate the following working hypothesis: 5-HT_2A_R is the dominant receptor governing respiratory control as evidenced by its stronger expression and constitutive activity. 5-HT_2B_R, being present in all respiratory nuclei analyzed and co-expressed with 5-HT_2A_R in many cells, although at a much reduced level, may act as a dose-dependent modulator. If more serotonin is released than needed to activate all 5-HT_2A_Rs, 5-HT_2B_Rs become activated. This would allow the system to regulate the respiratory rhythm by controlling serotonin release. Activation of spare 5-HT_2B_R has the strongest effect on respiratory frequency. This regulation could, at least in part, be due to Ca^2+^ signaling, as the presence of 5-HT_2B_R changes the kinetics of the Ca^2+^ signaling observed for both receptors alone, without altering the overall amount of mobilized calcium.
